# Single-dose pharmacokinetics and safety of azilsartan medoxomil in children and adolescents with hypertension as compared to healthy adults

**DOI:** 10.1007/s00228-015-1987-8

**Published:** 2016-01-04

**Authors:** Nicholas J. A. Webb, Thomas Wells, Max Tsai, Zhen Zhao, Attila Juhasz, Caroline Dudkowski

**Affiliations:** Department of Paediatric Nephrology, Royal Manchester Children’s Hospital, Central Manchester University Hospitals NHS Foundation Trust, Manchester Academic Health Science Centre, Manchester, M13 9WL UK; Institute of Human Development, Faculty of Medical and Human Sciences, University of Manchester, Manchester, UK; University of Arkansas for Medical Sciences, Arkansas Children’s Hospital, Little Rock, AR USA; Takeda Development Center Americas, Inc., Deerfield, IL USA; Takeda Development Centre Europe, Ltd, London, UK

**Keywords:** Angiotensin receptor blocker, Azilsartan medoxomil, Pediatric, Pharmacokinetics, Dosing

## Abstract

**Purpose:**

This open-label, multicenter, single-dose study characterized the pharmacokinetics and short-term safety of azilsartan medoxomil (AZL-M) in hypertensive pediatric subjects (12–16 years [cohort 1a; *n* = 9]; 6–11 years [cohort 2; *n* = 8]; 4–5 years [cohort 3; *n* = 3]).

**Methods:**

Model-based simulations were performed to guide dosing, especially in 1–5-year olds, who were difficult to enroll. AZL-M was dosed according to body weight (20–60-mg tablet, cohorts 1a and 2; 0.66 mg/kg granule suspension, cohort 3). In cohort 1, gender-matched healthy adults (cohort 1b; *n* = 9) received AZL-M 80 mg.

**Results:**

Exposure to AZL (active moiety of AZL-M), measured by dose-/body weight-normalized *C*_max_ and AUC_0–∞_, was ∼15–30 % lower in pediatric subjects versus adults. In simulations, exposure with 0.66 mg/kg AZL-M in pediatric subjects weighing 8–25 kg approximated to AZL-M 40 mg (typical starting dose) in adults. The simulations suggest that 25–50-kg subjects require half the adult dose (10–40 mg), whereas 50–100-kg subjects can use the same dosing as adults. Adverse events were mild in intensity, apart from one moderate event (migraine).

**Conclusions:**

This dosing strategy should be safe in pediatric patients, as AZL exposure would not exceed that seen in adults with the highest approved AZL-M dose (80 mg).

**Electronic supplementary material:**

The online version of this article (doi:10.1007/s00228-015-1987-8) contains supplementary material, which is available to authorized users.

## Introduction

Pediatric hypertension is a significant, frequently undiagnosed illness and is on the increase, particularly in obese patients [1–4]. Recent estimates suggest a prevalence of 3–5 % in children and adolescents [1, 5–7]. Beyond the challenges of diagnosis, pediatric hypertension can also be mismanaged due to lack of familiarity with appropriate therapies and concern over possible adverse effects [4].

Antihypertensive drug therapy has shown effectiveness in pediatric patients and is increasingly being employed [8–11]. Angiotensin II receptor blockers (ARBs) are one of the many options for pediatric hypertension [8–10]. Several clinical trials have demonstrated their efficacy and tolerability in children aged 1 year to adolescence [12–19].

Azilsartan medoxomil (AZL-M) is among the most effective ARBs tested to date in terms of blood pressure-lowering efficacy, and is approved for the treatment of hypertension in adults at a dose of 40–80 mg once daily (20–80 mg in the EU), alone or in combination with other antihypertensive agents [20–22]. AZL-M is a prodrug that is rapidly hydrolyzed during absorption in the gut to form its active moiety azilsartan (AZL) [20–23]. Based on previous pharmacokinetic (PK) analyses in adults, peak plasma concentrations of AZL are reached within 1.5–3 h after oral dosing and elimination half-life (*t*_½_) is approximately 11–12 h after administration of AZL-M [20, 23]. The volume of distribution of AZL is approximately 16 L [21]. AZL is eliminated via both renal clearance (∼2.3 mL/min) and hepatic metabolism, although neither mild-to-severe renal impairment nor mild-to-moderate hepatic impairment affect AZL exposure to any clinically relevant degree [20, 23–25]. The principle metabolite of AZL (M-II; formed by O-dealkylation via the cytochrome P450 2C9 isoform) and the main minor metabolite (M-I; formed by decarboxylation) are both pharmacologically inactive [20, 23].

To date, AZL-M has not been investigated in children. Selection of appropriate pediatric dosages requires understanding a drug’s PK profile across a suitable range of ages and body weights [26]. Therefore, we evaluated the PK and safety of AZL-M in hypertensive subjects aged 4–16 years and in healthy adults. In addition, model-based simulations were used to provide guidance on suitable dosing, especially in very young subjects (aged 1–5 years) with lower body weight.

## Methods

This phase 1, open-label, multicenter, single-dose study (ClinicalTrials.gov identifier: NCT01078376) comprised a screening period (days −28 to −2), a check-in period (day −1), a treatment period (days 1 to 2), and a follow-up period, which included a phone call on study days 6 and 15 (±1 day). The study was performed in the UK (three centers) and the USA (six centers). It was approved by applicable institutional review boards or ethics committees and conducted in accordance with the Declaration of Helsinki and Good Clinical Practice guidelines. All subjects, or their parents/legal guardians, gave written informed consent (and age-appropriate assent, where applicable) to participate.

### Study participants

Twenty hypertensive boys and girls aged 4–16 years enrolled in three separate cohorts as follows: nine subjects aged 12–16 years (adolescents; cohort 1a), eight subjects aged 6–11 years (cohort 2), and three subjects aged 4–5 years (cohort 3). The target number of eight subjects aged 1–5 years was originally planned for cohort 3, but we experienced recruitment difficulties. Cohort 1b included nine gender-matched healthy adults aged 18–45 years, inclusive. Eligible pediatric subjects were required to have a diagnosis of hypertension, with systolic blood pressure (SBP) and/or diastolic blood pressure (DBP) ≥95th percentile for gender/age/height [27]. For cohorts 1a and 2, subjects had to be within the weight range 20–100 kg at screening; for cohort 3, subjects had to weigh ≥8.0 kg. Adult participants had to weigh ≥50 kg, with a BMI of 18–32 kg/m^2^, and DBP 60–90 mmHg and SBP 100–140 mmHg at screening and check-in. Clinical laboratory results had to be within the reference range for the testing laboratory, unless results were deemed not clinically significant by the investigator.

Exclusion criteria for pediatric subjects included current treatment with >2 antihypertensive agents; sitting trough clinic SBP >15 mmHg or DBP >10 mmHg above the 99th percentile for gender/ age/height; renovascular disease, dialysis treatment, or active severe nephrotic syndrome; previous renal transplant (cohorts 1a and 2 only); and creatinine clearance <30 mL/min/1.73 m^2^. Exclusion criteria for all subjects included known hypersensitivity to ARBs; clinically relevant history of severe cardiovascular disease; malignant or accelerated hypertension; severe hepatic impairment; serum albumin <2.5 g/dL; glycosylated hemoglobin (HbA_1c_) >8.5 %; alanine aminotransferase (ALT) or aspartate aminotransferase (AST) >2 times the upper limit of normal, active liver disease, or jaundice; history of cancer not in remission for ≥5 years; and history of drug/alcohol abuse.

Pediatric subjects on ACE inhibitors and other ARBs were to withhold these medications from the morning of day −1 until the PK sample at 24 h was completed. Use of concomitant medications was not allowed during the study (except for occasional paracetamol/acetaminophen ≤1 g/ day for pediatric subjects and ≤2 g/day for adults), unless deemed necessary for medical emergency or approved on a case-by-case basis. For pediatric subjects only, concomitant medications for primary renal or urologic conditions or hypertension were allowed if their doses had been stable for ≥30 days prior to check-in (day −1) and they were not known potent inhibitors or inducers of any cytochrome P450 enzymes.

### Treatment allocation

All pediatric subjects received a single oral dose of AZL-M according to body weight. Subjects in cohort 3 (≤5 years) received AZL-M equivalent to 0.66 mg/kg body weight. Subjects in cohorts 1a and 2 (≥6 years) received AZL-M 20 mg (range 0.5–1.0 mg/kg) for body weights of 20 to <40 kg, AZL-M 40 mg (range 0.5–1.0 mg/kg) for 40 to <80 kg, or AZL-M 60 mg (range 0.6–0.75 mg/kg) for 80 to 100 kg. All adults received a single 80-mg AZL-M oral dose. In cohorts 1 and 2, the dose was administered as 1–2 tablets (20 and/or 40 mg); for cohort 3, the dose was administered as an oral liquid suspension of granules in water. The granule formulation (sachets containing 10 mg AZL-M for reconstitution) contained AZL-M, mannitol, fumaric acid, sodium hydroxide, hydroxypropyl cellulose, sucralose, grape flavor powder, and purified water. In a phase 1 bioequivalence study comparing AZL-M 20 mg granules versus tablets, the granule/tablet ratio for AZL AUC_0–∞_was 118 % and the 90 % CI was within the bioequivalence limits of 80–125 % (data on file). The AZL *C*_max_ ratio was 147 % (above the bioequivalence limit) due to relatively rapid appearance of AZL in the plasma compared with the tablet formulation (in adults, median *t*_max_ = 1.00 h for granules and 2.00 h for tablet) (data on file). This was not unexpected, as no dissolution step before oral absorption is involved with the reconstituted granule dosage form, and it is not considered clinically significant.

Subjects fasted for ≥4 h (cohorts 1 and 2) or ≥3 h (if able, cohort 3) prior to study drug administration and 1 h after.

### Sampling and bioanalytical methods

For cohorts 1 and 2, one blood sample (2 mL for children/adolescents, 6 mL for adults) was obtained predose and 0.25, 0.5, 1, 2, 4, 6, 8, 12, and 24 h postdose. For cohort 3, one blood sample (all 1 mL, except 2 mL for 0.25- and 1-h samples) was obtained predose and 0.25, 1, 6, 12, and 24 h postdose. Blood samples were collected into chilled 6-mL tubes containing potassium ethylene diamine tetra-acetic acid. Plasma was separated by centrifugation and samples stored at approximately −20 °C or lower. Urine samples (cohorts 1 and 2 only) were obtained, where possible, for the determination of concentrations of AZL and M-II in urine predose (single collection between −12 and 0 h), and 0–4, 4–8, 8–12, and 12–24 h postdose. Urine samples were stored at approximately 4 °C during the collection interval and, thereafter, as two 10-mL aliquots at approximately −20 °C or lower.

Concentrations of AZL and M-II in plasma and urine were determined using validated liquid chromatography-tandem mass spectrometry (LC-MS/MS) assays at Covance Laboratories, Madison, WI, USA. For plasma, 2 % acetic acid in acetonitrile solution with internal standard was added to samples for protein precipitation. For urine, 0.1 % acetic acid in methanol with internal standard was added to samples for a 1:6 dilution. After mixing, another aliquot of 0.1 % acetic acid in methanol was added. Liquid chromatography separation was obtained using a Chromolith SpeedROD RP-18e column (EMD Millipore; 50 × 4.6 mm). The mobile phase consisted of a gradient 0.1 % acetic acid in water/0.1 % acetic acid in methanol and was pumped through the column at 2 mL/min. For detection, an API 3000 or 4000 mass spectrometer (AB Sciex, Framingham, MA, USA) with positive ion electrospray in multiple-reaction monitoring mode was employed. The LC-MS/MS assay ranges for the detection of AZL and M-II in plasma were 10–5000 ng/mL and 2–1000 ng/mL, respectively. The range for both AZL and M-II in urine was 50–10,000 ng/mL.

### Pharmacokinetic and safety assessments

Pharmacokinetic variables derived from AZL and M-II concentrations in plasma included areas under the plasma concentration-time curve from 0–24 h postdose (AUC_0–24_) and 0 h–infinity (AUC_0–∞_); maximum observed concentration in plasma (*C*_max_); time to reach *C*_max_ (*t*_max_); terminal elimination rate constant (*λ*_z_ = negative slope of the log-linear regression of the natural logarithm concentration-time curve during the terminal phase); terminal elimination half-life (*t*_½_ = ln(2)/*λ*_z_); apparent oral clearance (CL/F = dose/AUC_0–∞_); and apparent volume of distribution during the terminal phase (V_z_/F = [CL/F]/*λ*_z_). CL/F and V_z_/F for AZL were calculated assuming 100 % conversion of AZL-M to AZL. Urine PK parameters included total amount excreted in urine from 0–24 h postdose (*Ae*_0–24_); fraction excreted in the urine (Fe = [*Ae*_0–24_/dose] × 100); and renal clearance (CL_*r*_ = Ae_0–24_/AUC_0–24_). Estimates of Fe for AZL and M-II were adjusted for molecular weight.

Pharmacokinetic parameters were derived using noncompartmental methods with WinNonlin® Professional Version 6.3 (Pharsight Corp., Mountain View, CA). Plasma and urine PK parameters for each cohort were summarized using descriptive statistics. Planned sample size was 24 pediatric subjects (8 in each cohort) and 8 adults. This sample size was determined primarily by clinical judgment, with consideration also given to past PK studies. It was expected to provide useful estimates of PK parameters and safety information in the specified pediatric populations.

Safety and tolerability parameters included adverse events, clinical laboratory tests (hematology, serum chemistry, urinalysis), vital signs, 12-lead ECG, and physical examination findings. Adverse events were monitored on the treatment day (day 1), upon study exit (day 2; 24 h after dosing), and at 6-/15-day follow-up via telephone. Laboratory tests, ECG, and vital signs were measured before treatment (on days −1 or 1) and after treatment on days 1 and/or 2. An independent data monitoring committee monitored PK and safety data during the trial. This included a review of data for all pediatric subjects in cohorts 1a and 2 and adult subjects in cohort 1b prior to initiating cohort 3.

### Model-based PK simulation

A two-compartment model with first-order absorption and elimination was used previously to describe AZL’s pharmacokinetics [28]. In line with previous pediatric models [29–33], this model was modified slightly using allometric adjustment of CL/F and V_z_/F according to body weight, as follows:

$$ {P}_{\mathrm{i}}={P}_{\mathrm{pop}}\cdot {\left(\frac{{\mathrm{WT}}_i}{{\mathrm{WT}}_{\mathrm{reference}}}\right)}^b $$

where *P*_i_ is the individual PK parameter, *P*_pop_ is the population PK parameter, WT_i_ is the individual body weight, WT_reference_ is the standard population body weight of 70 kg, and *b* represents an allometric power function describing the relationship between weight and the PK parameter (*b* = 0.75 for systemic clearance and *b* = 1.0 for volume of distribution). Similar results were observed in initial models considering body surface area (BSA) instead of body weight.

Intersubject variability was estimated on model parameters, based on a lognormal distribution with a mean = 0 and variance = *ω*^2^. In addition to the effect of body weight on CL/F and V_z_/F in the base model, other covariate effects (age, race, gender, and glomerular filtration rate [eGFR; estimated from serum creatinine using the Schwartz formula;]) [34] were also evaluated, but did not improve the model fit significantly so they were not included in the final model.

A separate effect on the absorption rate constant (*k*_a_) was estimated for cohort 3 to account for different formulations (tablet versus granules) used in this study. The faster absorption rate associated with granules relative to tablets is consistent with the noncompartmental analysis results of a bioequivalence study in adults (data on file). Similarly, a separate effect on relative bioavailability (*F*) was included to allow for the slightly higher bioavailability seen with the granule formulation in that study.

The population PK analysis and simulations were conducted using the first-order conditional estimation method with η-ε interaction in NONMEM Version 7.1.2 operating on a grid cluster. The model was evaluated using a visual predictive check; a nonparametric bootstrap re-sampling technique was also performed to assess its robustness by comparing model parameter estimates to the distribution of those obtained from bootstrap runs that converged successfully.

Model-based simulations were performed to project exposure in pediatric subjects weighing 25–100 kg. A pediatric population whose weights were uniformly distributed within the specified range was generated and equally allocated to fixed AZL-M doses of 10, 20, 40, and 80 mg. Simulations were also performed to project exposure in young subjects (1–5 years) weighing <25 kg because we had originally planned to enroll subjects as young as 1 year old to have data to inform that population, but we experienced recruitment difficulties. Since no data were collected in subjects less than 4 years of age, it was assumed that this younger population demonstrated similar pharmacokinetics to that of older children. A population of pediatric subjects whose weights were uniformly distributed within the range of 8–25 kg was generated and equally allocated to weight-based AZL-M doses from 0.05–1.5 mg/kg.

## Results

### Subject characteristics

Fifty-nine subjects were screened, and 29 were enrolled (20 pediatric, 9 adults). Cohorts 1 and 2 met or exceeded their planned sample size. Although recruitment for cohort 3 lasted 22 months, it was only possible to enroll three of the planned eight subjects. The youngest was 4 years old, and the lightest weighed 13.9 kg. Due to these enrollment difficulties, it was decided to discontinue the study without achieving the planned sample size in cohort 3 and to use PK modeling to determine the appropriate dosing in children aged 1–5 years for future studies. Demographic characteristics for the three cohorts are summarized in Supplementary Table S1. Mean SBP/DBP was 130/69, 116/14, and 104/64 mmHg in pediatric cohorts 1a, 2, and 3, respectively, and 114/68 in healthy adults (cohort 1b). Eight of nine adolescent subjects in cohort 1b, all eight children in cohort 2, and the three children in cohort 3 received a single body weight-adjusted dose of AZL-M. The exception was one subject (adolescent in cohort 1a) with a check-in weight of 70.5 kg who received AZL-M 60 mg rather than 40 mg in error; the subject did not experience any adverse events. The individual per kilogram doses received in cohorts 1a, 2, and 3 ranged from 0.51–0.97 mg/kg (Supplementary Table S2). All nine adults received a single AZL-M 80-mg dose, with individual per kilogram doses of 0.92–1.53 mg/kg (mean 1.10 mg/kg). All available data for the 29 subjects were included in the safety and PK analyses. One subject in cohort 1a had only two (instead of nine) postdose PK samples and was not included in the PK parameter summaries; an additional subject was recruited to meet the minimum number (8) of evaluable subjects for PK analysis.

### Plasma pharmacokinetics

**Fig. 1:**
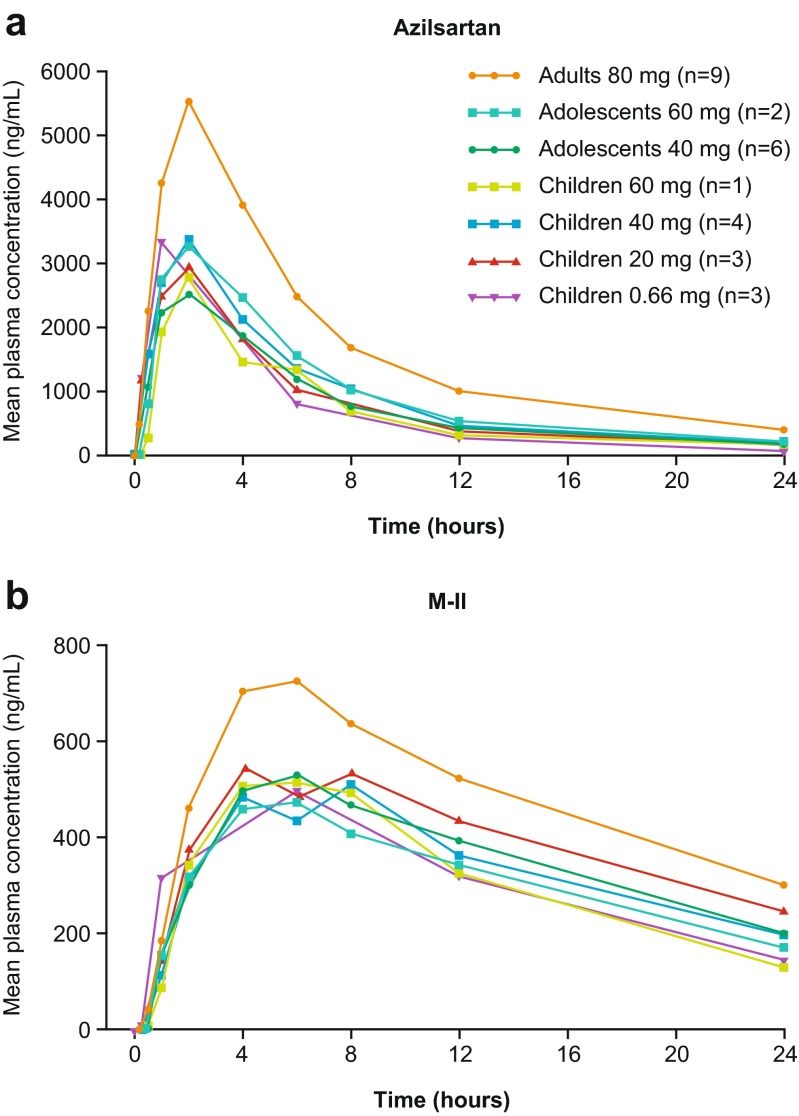
Mean plasma concentration-time profiles of AZL and M-II

Individual concentration-time profiles of AZL and M-II grouped by cohort and AZL-M dose received are shown in Fig. 1. Pharmacokinetic parameter estimates are shown in Supplementary Table S2. Median *t*_max_ for AZL was 1 h postdose in the three children receiving AZL-M granules and approximately 2 h postdose in all other subjects; median *t*_max_ values for M-II ranged from 4–6 h postdose across all doses (Supplementary Table S2). In general, pediatric subjects had approximately 50 % lower *C*_max_ and AUC_0–∞_ values of AZL (without normalization for dose and body weight adjustment) than the healthy adults (80-mg dose), reflecting the lower per kilogram doses received (Supplementary Table S2). Dose- and body weight-normalized *C*_max_ and AUC_0–∞_ values of AZL were 15–30 % lower than in healthy adults, the exception being *C*_max_ with AZL-M 0.66 mg/kg in cohort 3 (mean 5240 [ng/mL]/[mg/kg]), where values were similar to those in adults (Table 1). Furthermore, within cohort 2, children receiving AZL-M 40 mg had a higher mean adjusted *C*_max_ (5404 [ng/mL]/[mg/kg]). Overall, intersubject variability for PK parameters of AZL was low. Pediatric subjects had dose- and body weight-normalized *C*_max_ and AUC_0–∞_ values of M-II similar to those of adults. In general, mean *t*_½_ values for AZL and M-II were lower in pediatric subjects than in healthy adults (Supplementary Table S2).

**Table 1:** AZL-M dose- and body weight-normalized exposure and body weight-adjusted plasma PK parameter estimates for AZL and M-II

Parameter	Cohort 1				Cohort 2				Cohort 3
	Healthy adults	Adolescents (12 to 16 years)			Children (6 to 11 years)				Children (4 to 5 years)
Dose	80 mg (*n* = 9)	All doses (*n* = 8)	60 mg (*n* = 2)	40 mg (*n* = 6)	All doses (*n* = 8)	60 mg (*n* = 1)	40 mg (*n* = 4)	20 mg (*n* = 3)	0.66 mg/kg (*n* = 3)
*AZL*									
AUC_0-∞_, [ng⋅h/mL]/[mg/kg]	42,276 (36)	30,687 (14)	35,891 (3)	28,952 (12)	29,337 (39)	22,360 (NA)	34,001 (36)	25,445 (45)	28,180 (33)
*C*_max_, [ng/mL]/[mg/kg]	5251 (26)	4031 (19)	4523 (19)	3867 (19)	4600 (39)	3794 (NA)	5404 (41)	3797 (25)	5240 (21)
CL/F, L/h/kg	0.02 (37)	0.03 (14)	0.02 (3)	0.03 (12)	0.03 (33)	0.04 (NA)	0.03 (31)	0.04 (36)	0.03 (28)
V_z_/F, L/kg	0.22 (30)	0.24 (18)	0.25 (5)	0.23 (22)	0.23 (23)	0.26 (NA)	0.21 (20)	0.26 (24)	0.19 (32)
*M-II*									
AUC_0-∞,_ [ng⋅h/mL]/[mg/kg]	17,640 (39)	18,315 (28)	14,640 (12)	19,540 (28)	17,073 (49)	12,097 (NA)	17,387 (65)	18,313 (32)	14,793 (45)
*C*_max,_ [ng/mL]/[mg/kg	653 (17)	799 (35)	683 (37)	837 (35)	768 (40)	694 (NA)	829 (54)	711 (10)	762 (39)

Data are mean (%CV)

*AUC*_*0-∞*_ area under the plasma concentration-time curve from time 0– infinity (adjusted for dose and body weight), *CL/F* apparent oral clearance (adjusted for body weight), *C*_*max*_ maximum plasma concentration (adjusted for dose and body weight), *V*_*z*_ */F* apparent volume of distribution during the terminal phase (adjusted for body weight)

**Fig. 2:**
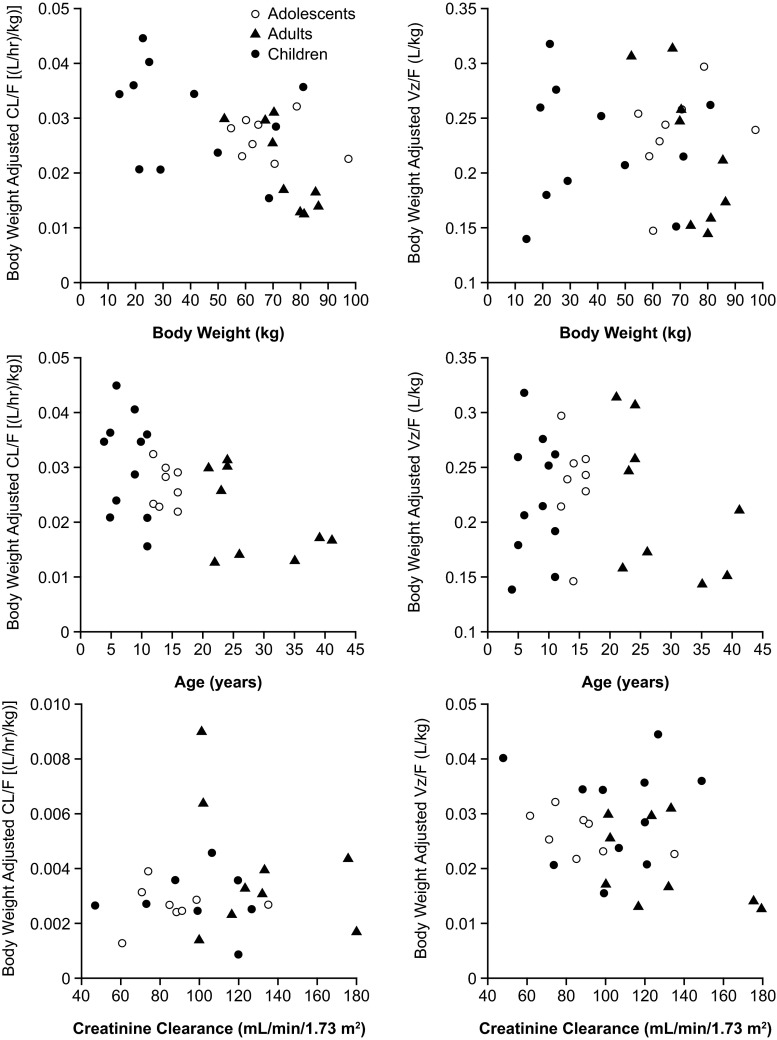
Scatter plots of body weight-adjusted CL/F and V_z_/F values for AZL versus body weight (*top*) and age (*middle*) and body weight-adjusted CL/F and CL_*r*_ versus creatinine clearance (*bottom*)

Mean body weight-normalized CL/F and V_z_/F values for AZL were slightly higher in pediatric subjects compared with healthy adults (Table 1). From the scatter plots of body weight-adjusted CL/F values for AZL versus body weight, it was apparent that the dosing scheme for pediatric subjects did not completely correct for body weight, as subjects with lower body weight tended to have a higher CL/F (Fig. 2). Similarly, from the scatter plot of body weight-adjusted CL/F values for AZL versus age, younger subjects tended to have a higher CL/F (Fig. 2). There were no observed trends in weight-adjusted V_z_F values for AZL versus body weight or age, or in weight-adjusted CL/F values for AZL versus creatinine clearance (Fig. 2).

### Urinary pharmacokinetics

The mean Fe and body weight-corrected CL_*r*_ values of AZL in pediatric subjects ranged from 8 to 11 % and 0.0026 to 0.0035 L/h/kg, respectively, and were lower than the values in healthy adults (16.7 % and 0.0039 L/h/kg, respectively) (Supplementary Table S3). A scatter plot of body weight-adjusted CL_*r*_ values for AZL versus creatinine clearance did not reveal any apparent trend (Fig. 2). The mean Fe and body weight-corrected CL_*r*_ values for M-II in pediatric subjects ranged from 7 to 10 % and 0.0044 to 0.0074 L/h/kg, respectively, and were also lower than the values in healthy adults (11.0 % and 0.0080 L/h/kg, respectively) (Supplementary Table S3). The mean Ae_0–24_ values of AZL and M-II reflected the differing doses in pediatric and adult subjects.

### Modeling and simulation

The modified PK model adequately described the concentration-time data from this study (Supplementary Fig. S1). The parameter estimates and their associated precision (%SEM) are listed in Supplementary Table S4. The PK parameters were generally estimated with good precision and consistent with those obtained following bootstrap analysis. CL/F and *k*_a_ intersubject variability was 0.116 (34 %CV) and 0.490 (70 %CV), respectively. An effect of gender on CL/F was significant at the *p* = 0.05 level, but was not clinically relevant (∼25 % difference between male and female), with only a minor (∼10 %) reduction in the intersubject variability of CL/F; consequently, this covariate effect was not retained in the model. No other covariate effects were noted, except for body weight and formulation effects, which were included as part of the base structural model.

**Fig. 3:**
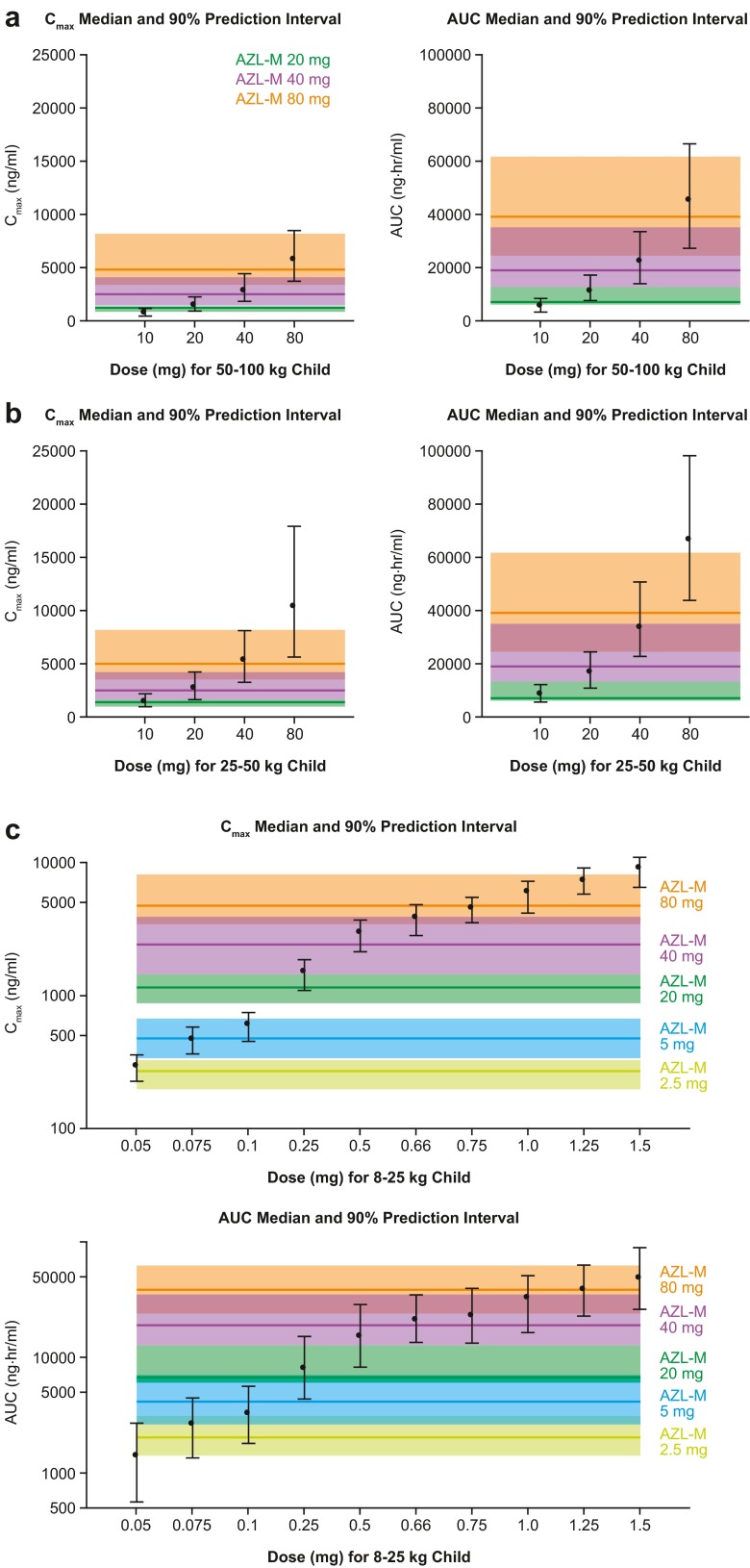
Simulated exposures to AZL in **a** subjects 6–16 years of age and body weight 50–100 kg, **b** subjects 6–16 years of age and body weight 25–50 kg, and **c** subjects 1–5 years of age and body weight 8–25 kg. The *point and error bars* represent median and 5th and 95th percentiles of *C*_max_ and AUC values predicted in pediatric subjects. Similarly, the *solid line and shaded regions* median and 5th and 95th percentiles of *C*_max_ and AUC values, based on observed data in healthy adults pooled from phase I studies (20, 40 , and 80 mg doses are based on studies with AZL-M [TAK-491]; lower doses are based on studies with AZL [TAK-536]). AZL 2.5 and 5 mg are approximately similar to AZL-M 5 and 10 mg, respectively, based on AUC or total exposure of AZL

The model-predicted mean *C*_max_ and AUC values stratified by cohort and dose were generally very consistent with those observed from the study (Supplementary Table S5). Based on the visual predictive check, the predicted median values stratified by cohort were generally consistent with the central tendency of the observed data (Supplementary Fig. S2). Based on the predicted median and 90 % range of *C*_max_ and AUC values, pediatric subjects weighing 50–100 kg had approximately the same total (i.e., not body weight-adjusted) exposure to AZL as healthy adults at the same fixed AZL-M dose (Fig. 3a). However, pediatric subjects weighing 25–50 kg had approximately double the total AZL exposure as adults at the same fixed AZL-M dose (Fig. 3b).

The projected steady-state AZL exposure following repeated AZL-M doses of 0.05–1.5 mg/kg in pediatric subjects with body weights of 8–25 kg is shown in Fig. 3c, with reference to observed PK data for AZL-M or AZL in adults pooled from the phase 1 program. These reference values are obtained from studies with AZL 2.5 and 5 mg (approximately equivalent to AZL-M 5 and 10 mg, respectively) and AZL-M 10–80 mg. Exposure to AZL (AUC) with a 0.66-mg/kg AZL-M dose in pediatric subjects was predicted to be comparable to that with a 40-mg AZL-M dose in adults.

### Safety and tolerability

No treatment-emergent serious adverse events (AEs) were reported. Overall, 10 of 29 subjects (34.5 %) experienced a total of 15 AEs during the study. Five subjects (55.6 %) in cohort 1a experienced ≥1 AE (2 abdominal discomfort, 2 headache, 1 dizziness, 1 migraine, 1 hematoma). Three subjects (37.5 %) in cohort 2 experienced ≥1 AE (1 venipuncture site pain, 1 sinusitis, 1 arthralgia, 1 headache, 1 oropharyngeal pain). No subjects in cohort 3 experienced an AE. Two adult subjects (22.2 %) experienced an AE (1 sinus bradycardia, 1 infected bites). One subject experienced an AE of moderate intensity (migraine in cohort 1a); all other AEs were mild. Only two subjects (both in cohort 1a) experienced an AE considered to be related to study medication (headache and migraine). There were no reports of post-treatment serious AEs, of clinically significant ECG findings, or of AEs pertaining to laboratory parameters or vital signs.

As expected, treatment with AZL-M was associated with a reduction in BP. At study exit, mean SBP/DBP was 125/70, 112/66, and 101/57 mmHg in pediatric cohorts 1a, 2, and 3, respectively, and 101/65 in healthy adults (cohort 1b). One adult experienced very low SBP (76 mmHg) at 24 h postdosing, as did one child in cohort 3 at 3 and 10 h postdose (74 and 75 mmHg, respectively), but these events resolved.

## Discussion

The current study aimed to characterize the PK profile, safety, and tolerability of a single dose of AZL-M in pediatric subjects with hypertension aged 1–16 years and healthy adults. The appearance of AZL in the plasma was generally similar across the subject groups (children 6–12 years versus adolescents 13–16 years versus healthy adults), as measured by time to maximum plasma concentration (median 2 h). In the three younger children receiving AZL-M granules, absorption appeared to be faster (median *t*_max_ = 1 h). This likely reflects the differences in the formulation, rather than younger age, as a previous study has shown more rapid absorption of the granule versus tablet formulation in adults (data on file). AZL exposure (*C*_max_ and AUC_0–∞_) at all AZL-M doses tested was approximately 50 % lower in pediatric subjects with hypertension relative to the values for 80 mg AZL-M in healthy adults, and remained around 15–30 % lower after dose and body weight normalization. Thus, the initial per kilogram body weight-adjusted AZL-M doses chosen for investigation in children in the current study (20–60 mg for children weighing 25–100 kg; 0.66 mg/kg for children 1–5 years of age and weighing ≥8 kg) achieved lower per kilogram levels of AZL exposure for each milligram of AZL-M dosed than did the highest approved dose in adults (80 mg).

The decreased exposures to AZL in children are in agreement with the trends observed in the scatter plots. Although body weight-adjusted CL/F and V_z_/F were relatively similar in the different cohorts, trends were observed between individual body weight-adjusted CL/F values for AZL and body weight, and individual body weight-adjusted CL/F values for AZL and age: a subject with a lower body weight or a younger subject had a higher body weight-adjusted CL/F. A similar trend using body weight-adjusted dosing has also been observed with the ARB olmesartan [35]. The trends with body weight and age are consistent with the allometric relationship described previously, whereby clearance per kilogram of body weight tends to decrease with increasing age and weight from the age of 1 year up to adolescence [29, 30]. The results suggest that a dose adjustment approach simply based on dose per kilogram body weight does not completely correct for developmental differences in clearance. The median *t*_max_ and the mean dose- and body weight-normalized *C*_max_ and AUC_0–∞_ values of the M-II metabolite were similar in pediatric subjects and adults.

The difficulties inherent in conducting pediatric PK studies are widely recognized [29, 32, 36]. Recruiting very young children, in particular, is challenging especially to single-dose studies such as this, where there are no perceived benefits to participation. Parents are understandably concerned about consenting their child to confinement to hospital and repeated blood sampling [29]. In this particular study, given the relatively long half-life of azilsartan, the study protocol mandated that study participants remained in hospital overnight rather than allowing children to go home between the 12- and 24-h PK blood samplings; this issue alone resulted in a number of parents declining consent. We recruited the full planned complement of subjects ≥6 years of age, but only three of the planned eight subjects aged ≤5 years, the youngest 4 years of age and the lightest weighing 13.9 kg.

Due to these recruitment difficulties, individual PK concentration data obtained were used to develop a population PK model that evaluated the applicability of the AZL-M 0.66-mg/kg dose across the body weight range of 8–25 kg. The current model incorporated a more refined correction for body weight based on a well-established allometric scaling factor (in which clearance is standardized to a 70-kg person using a power coefficient of 0.75) [29, 30], as well as adjustments for the shorter *t*_max_ and slightly higher bioavailability with the granule formulation relative to the tablet formulation. As initial models considering BSA instead of body weight provided similar results, adjustment for body weight was considered sufficient. Although developmental differences other than body weight might also have influenced AZL clearance, no additional covariate effects, including age, race, gender, and eGFR, were evident after weight had been accounted for in the modeling analysis. Body weight (along with age) has also been shown to influence AZL clearance in adults, according to a model-based population PK analysis [28]. The current model-based results indicate that the weight-based dosing regimen of 0.66 mg/kg was suitable for the weight range of 8–25 kg, as the predicted exposure in this group was approximately equivalent to (and did not exceed) that observed previously after AZL-M 40 mg (a typical therapeutic starting dose) in adults, and therefore, should not pose a safety risk. An AZL-M dose level of 1–1.25 mg/kg in these pediatric subjects would be equivalent to the highest approved AZL-M dose in adults (80 mg), whereas an AZL-M dose level of 0.05–0.075 mg/kg would be approximately equivalent to a minimally effective AZL-M dose of 5 mg in adults (data on file).

Model-based simulations were also performed to evaluate the applicability of the body weight-adjusted AZL-M tablet doses used in pediatric subjects in the weight ranges 25–50 kg and 50–100 kg. According to the simulations, pediatric subjects with a body weight of 50–100 kg would have AZL exposure similar to that in healthy adults at the same AZL-M dose, whereas pediatric subjects weighing 25–50 kg would have approximately double the exposure of adults at the same dose. However, even at an AZL-M dose of 80 mg, AZL exposure in 25–50-kg children would still not exceed the maximum exposure previously evaluated for safety and tolerability in adults (at doses up to 320 mg). A similar model-based approach has previously been employed to support dosing with the ARB olmesartan in children aged 6–16 years [37].

Given that CYP2C9 is the main enzyme involved in AZL elimination, any age-dependent variability in the expression and activity profiles of CYP2C9 might be a major factor influencing AZL dosing in very small children. It has previously been shown that, from birth to 5 months of age, CYP2C9 protein and activity levels vary 35-fold, potentially making any dosing prediction difficult. However, from 5 months to 18 years, significantly less variability was observed [38]. Since renal maturation is not complete until 1 year after birth [39], the regulatory agencies advised against the enrolment of infants under 12 months of age into this study. The lower rate of CYP2C9 variability in children over 12 months of age, the target population for AZL/AZL-M, effectively eliminates this as a potential problem.

Single-dose administration of AZL-M to pediatric subjects with hypertension and healthy adult subjects was generally well tolerated at the doses used here. Interpretation of these results is limited by the small number of subjects and events, although no subject discontinued from the study and no post-treatment serious AEs were reported. Previous studies with other ARBs in hypertensive children aged 1–17 years have demonstrated safety and tolerability profiles similar to placebo and similar to those observed in adults [12–19, 35, 40, 41]. Based on the current clinical findings, we would expect a similarly favorable safety profile for AZL-M in hypertensive children aged ≥1 year.

## Conclusions

Despite a higher AZL clearance per kilogram of body weight, pediatric subjects with lower body weights may still require lower doses of AZL-M to achieve similar AZL exposures per kilogram compared with adults. The current weight-based dosing regimen of 0.66 mg/kg (using the granule formulation) is suitable for pediatric subjects with a body weight of 8–25 kg and should not pose any safety risk based on predicted exposure (approximately equivalent to a 40-mg dose in adults). Children weighing 25–50 kg may require only half the dose of adults (10–40 mg QD), whereas those weighing 50–100 kg can use the same dosing as adults (20–80 mg QD). Based on the data generated in the current study, these doses should be safe in pediatric patients. However, their antihypertensive efficacy and safety profile in the pediatric population remains to be determined in clinical trials.

## Electronic supplementary material

Supplementary Fig. S1 Goodness-of-Fit Plots for the Model-Based AZL PK Simulation Observations are concentration values (ng/mL); blue line shows trend line; red line shows unity line. (JPG 107 kb)

High resolution image (EPS 1.78 mb)

Supplementary Fig. S2 Visual Predictive Check for the Model-Based PK Simulation Observations are concentration values (ng/mL) (JPG 92 kb)

High resolution image (EPS 1.15 mb)

Table S1 (DOC 37 kb)

>Table S2 (DOC 49 kb)

Table S3 (DOC 40 kb)

Table S4 (DOC 34 kb)

Table S5 (DOC 46 kb)
