# Supplementary figures and images for: Single-dose pharmacokinetics and safety of azilsartan medoxomil in children and adolescents with hypertension as compared to healthy adults
Source: Eur J Clin Pharmacol. 2016 Jan 4;72:447–57. doi: 10.1007/s00228-015-1987-8 (PMC4792355; doi:10.1007/s00228-015-1987-8)

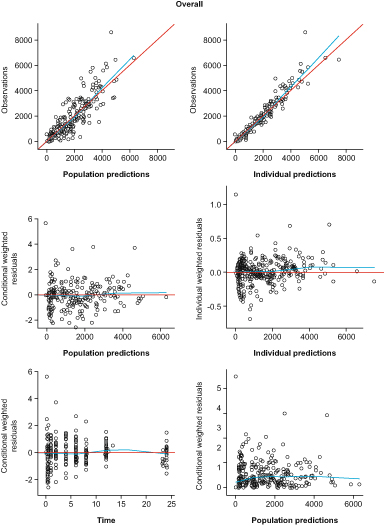

Supplement: Supplementary file 1 — Goodness-of-Fit Plots for the Model-Based AZL PK Simulation Observations are concentration values (ng/mL); blue line shows trend line; red line shows unity line. (JPG 107 kb) [file 228_2015_1987_Fig4_ESM.jpg]

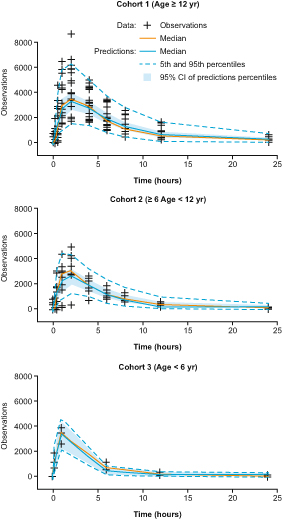

Supplement: Supplementary file 3 — Visual Predictive Check for the Model-Based PK Simulation Observations are concentration values (ng/mL) (JPG 92 kb) [file 228_2015_1987_Fig5_ESM.jpg]
