# Supplementary material for: Single-dose pharmacokinetics and safety of azilsartan medoxomil in children and adolescents with hypertension as compared to healthy adults
Source: Eur J Clin Pharmacol. 2016 Jan 4;72:447–57. doi: 10.1007/s00228-015-1987-8 (PMC4792355; doi:10.1007/s00228-015-1987-8)
Supplement: Supplementary file 5 — (DOC 37 kb) [file 228_2015_1987_MOESM3_ESM.doc]

**Supplemental Table S1.** Demographic Characteristics

| **Demographic variable** | **Cohort 1** | | **Cohort 2** | **Cohort 3** |
| --- | --- | --- | --- | --- |
|  | **Healthy adult matches** | **Adolescents (12 to 16 years)** | **Children (6 to 11 years)** | **Children (4 to 5 years)** |
| N | 9 | 9 | 8 | 3 |
| Gender, Female/Male (n) | 2/7 | 2/7 | 5/3 | 2/1 |
| Race, White/Black/Asian (n) | 8/1/0 | 7/2/0 | 5/2/1 | 1/2/0 |
| Age, years (mean ± SD) | 28.3 ± 7.8 | 14.2 ± 1.6 | 9.1 ± 2.1 | 4.7 ± 0.6 |
| BMI, kg/m^2^ (mean ± SD) | 25.1 ± 3.3 | 27.2 ± 6.6 | 24.3 ± 8.2 | 15.7 ± 0.6 |
| Body weight, kg (mean ± SD) [min, max] | 74.6 ± 11.2 [53.2, 90.5] | 71.7 ± 15.5  [55.6, 96.5] | 48.5 ± 22.5 [22.4, 80.0] | 18.3 ± 4.0 [13.9, 21.8] |
| SBP, mmHg (mean ± SD) | 114 ± 8 | 130 ± 9 | 116 ± 11 | 104 ± 7 |
| DBP, mmHg (mean ± SD) | 68 ± 7 | 69 ± 10 | 68 ± 14 | 64 ± 10 |
