# Supplementary material for: Single-dose pharmacokinetics and safety of azilsartan medoxomil in children and adolescents with hypertension as compared to healthy adults
Source: Eur J Clin Pharmacol. 2016 Jan 4;72:447–57. doi: 10.1007/s00228-015-1987-8 (PMC4792355; doi:10.1007/s00228-015-1987-8)
Supplement: Supplementary file 6 — (DOC 49 kb) [file 228_2015_1987_MOESM4_ESM.doc]

**Supplemental Table S2.** Plasma PK Parameter Estimates for AZL and M-II

| **Parameter** | **Cohort 1** | | | |  | **Cohort 2** | | |  | **Cohort 3** |
| --- | --- | --- | --- | --- | --- | --- | --- | --- | --- | --- |
|  | **Healthy adults** |  | **Adolescents (12 to 16 years)** | |  | **Children (6 to 11 years)** | | |  | **Children (4 to 5 years)** |
| **Dose** | **80 mg (n=9)** |  | **60 mg (n=2)** | **40 mg (n=6)** |  | **60 mg (n=1)** | **40 mg (n=4)** | **20 mg (n=3)** |  | **0.66 mg/kg (n=3)** |
| Dose/BW, mg/kg | 1.1 [0.9, 1.5] |  | 0.7 [0.6, 0.9] | 0.6 [0.5, 0.7] |  | 0.7 [NA] | 0.7 [0.6, 1.0] | 0.8 [0.7, 0.9] |  | 0.6 [0.6, 0.7] |
| ***AZL*** |  |  |  |  |  |  |  |  |  |  |
| AUC_0-_, ngh/mL | 44,820 (26) |  | 26,411 (25) | 18,686 (20) |  | 16,563 (NA) | 23,792 (26) | 19,543 (32) |  | 17,771 (30) |
| *C*_max_, ng/mL | 5,699 (24) |  | 3,245 (3) | 2,512 (28) |  | 2,810 (NA) | 3,858 (35) | 2,960 (12) |  | 3,320 (20) |
| *t*_max_, h | 2.0 [2.0, 4.0] |  | 2.0 [2.0, 2.0] | 2.0 [1.0, 2.0] |  | 2.0 [NA] | 2.0 [1.1, 6.0] | 2.0 [2.0, 2.0] |  | 1.0 [1.0, 1.0] |
| *t*_½_, h | 7.4 (15) |  | 7.7 (8) | 5.8 (20) |  | 5.1 (NA) | 5.8 (13) | 5.4 (17) |  | 4.6 (35) |
| CL/F, L/h | 1.5 (27) |  | 1.9 (25) | 1.8 (23) |  | 2.9 (NA) | 1.4 (30) | 0.9 (27) |  | 0.5 (25) |
| V_z_/F, L | 15.8 (21) |  | 20.7 (18) | 14.8 (33) |  | 21.2 (NA) | 11.6 (21) | 6.5 (13) |  | 3.6 (43) |
| ***M-II*** |  |  |  |  |  |  |  |  |  |  |
| AUC_0-_, ngh/mL | 19,188 (35) |  | 10,596 (11) | 12,532 (31) |  | 8,961 (NA) | 11,387 (48) | 14,230 (24) |  | 9,477 (49) |
| *C*_max_, ng/mL | 736 (33) |  | 480 (15) | 535 (37) |  | 514 (NA) | 561 (38) | 561 (7) |  | 488 (43) |
| *t*_max_, h | 6.0 (4.0, 8.0) |  | 5.0 (4.0, 6.0) | 6.0 (4.0, 6.1) |  | 6.0 (NA) | 4.0 (4.0, 8.0) | 4.0 (4.0, 8.0) |  | 6.0 (6.0, 6.0) |
| *t*_½_, h | 16.6 (41) |  | 12.8 (23) | 14.0 (17) |  | 8.5 (NA) | 11.6 (29) | 14.0 (21) |  | 10.4 (27) |

Data are mean (%CV), except for dose/body weight, which is mean [min, max] and *t*_max_, which is median [min, max]

AUC_0-_, area under the plasma concentration-time curve from time 0 to infinity; BW, body weight; CL/F, apparent oral clearance; *C*_max_, maximum plasma concentration; NA, not applicable; t_½_, terminal elimination half-life; *t*_max_, time to reach *C*_max_; V_z_/F, apparent volume of distribution during the terminal phase
