# Supplementary material for: Single-dose pharmacokinetics and safety of azilsartan medoxomil in children and adolescents with hypertension as compared to healthy adults
Source: Eur J Clin Pharmacol. 2016 Jan 4;72:447–57. doi: 10.1007/s00228-015-1987-8 (PMC4792355; doi:10.1007/s00228-015-1987-8)
Supplement: Supplementary file 7 — (DOC 40 kb) [file 228_2015_1987_MOESM5_ESM.doc]

**Supplemental Table S3.** Urine PK Parameter Estimates for AZL and M-II

| **Parameter** | **Cohort 1** | | | |  | **Cohort 2** | | |
| --- | --- | --- | --- | --- | --- | --- | --- | --- |
|  | **Healthy adult  matches** |  | **Adolescents (≥12 to <17 years)** | |  | **Children (≥6 to <12 years)** | | |
| **Dose** | **80 mg (n=9)** |  | **60 mg (n=3)^a^** | **40 mg (n=6)** |  | **60 mg (n=1)** | **40 mg (n=4)** | **20 mg (n=3)** |
| ***AZL*** |  |  |  |  |  |  |  |  |
| Fe, % | 16.7 (44) |  | 9.5 (23) | 9.0 (32) |  | 9.6 (NA) | 11.4 (58) | 7.9 (46) |
| CL*_r_*, L/h/kg | 0.0039 (61) |  | 0.0026 (0) | 0.0026 (33) |  | 0.0035 (NA) | 0.0028 (56) | 0.0026 (4) |
| Ae_0–24_, mg | 10.7 (44) |  | 4.6 (23) | 2.9 (32) |  | 4.6 (NA) | 3.6 (58) | 1.3 (46) |
| ***M-II*** |  |  |  |  |  |  |  |  |
| Fe, % | 11.0 (78) |  | 6.5 (20) | 9.1 (56) |  | 9.9 (NA) | 9.2 (43) | 9.9 (19) |
| CL*_r_*, L/h/kg | 0.0080 (72) |  | 0.0044 (0) | 0.0054 (47) |  | 0.0074 (NA) | 0.0066 (41) | 0.0063 (5) |
| Ae_0–24_, mg | 6.6 (78) |  | 2.9 (20) | 2.7 (56) |  | 4.4 (NA) | 2.8 (43) | 1.5 (19) |

Data are mean (%CV)

Ae_0–24_: total amount of AZL or M-II excreted in urine from 0–24 h postdose; CL*_r_*, renal clearance (adjusted for body weight); Fe, fraction of initial AZL-M dose excreted in the urine as AZL or M-II (adjusted for molecular weight); NA, not applicable

^a^n=2 for CL*_r_*
