# Supplementary material for: Single-dose pharmacokinetics and safety of azilsartan medoxomil in children and adolescents with hypertension as compared to healthy adults
Source: Eur J Clin Pharmacol. 2016 Jan 4;72:447–57. doi: 10.1007/s00228-015-1987-8 (PMC4792355; doi:10.1007/s00228-015-1987-8)
Supplement: Supplementary file 8 — (DOC 34 kb) [file 228_2015_1987_MOESM6_ESM.doc]

**Supplemental Table S4.** Parameter Estimates and Standard Errors From the AZL Base Structural Model in the Model-Based PK Simulation

| Parameter | Final Parameter Estimate | | Interindividual Variability / Residual Variability | |
| --- | --- | --- | --- | --- |
|  | Typical Value | %SEM | Magnitude | %SEM |
| Systemic clearance [CL/F] (L/h) | 1.4 | 8.6 | 34.1 %CV | 42.0 |
| Volume of distribution [V2/F] (L) | 8.4 | 4.0 | NE | NE |
| Absorption rate constant [KA] (1/h) | 1.0 | 24.1 | 70.0 %CV | 36.6 |
| Distribution clearance [Q/F] (L/h) | 0.6 | FIXED | NE | NE |
| Peripheral volume of distribution [V3/F] (L) | 15.5 | FIXED | NE | NE |
| Absorption lag time [ALAG1] (h) | 0.2 | FIXED | NE | NE |
| Ratio of Additive to Proportional Residual Error | 488 | 8.3 | NE | NE |
| Formulation on KA | 4.9 | 66.0 | NE | NE |
| Formulation on Relative Bioavailability [F] | 1.2 | FIXED | NE | NE |
| Residual Error | 0.04 | 14.7 | NE | NE |
| Minimum value of the objective function = 3339.5 | | | | |
| NA: Not Available; NE: Not Estimated; %SEM: percent standard error of the mean; %CV: percent coefficient of variation | | | | |
