# Supplementary material for: Single-dose pharmacokinetics and safety of azilsartan medoxomil in children and adolescents with hypertension as compared to healthy adults
Source: Eur J Clin Pharmacol. 2016 Jan 4;72:447–57. doi: 10.1007/s00228-015-1987-8 (PMC4792355; doi:10.1007/s00228-015-1987-8)
Supplement: Supplementary file 9 — (DOC 46 kb) [file 228_2015_1987_MOESM7_ESM.doc]

**Supplemental Table S5.** Observed and Model-Predicted Mean AZL *C*_max_ and AUC Values

| Subject Group/ |  | *C*_max_ | AUC | *C*_max_ | AUC |
| --- | --- | --- | --- | --- | --- |
| Regimen | N | (ng/mL) | (ng⋅h/mL) | (ng/mL) | (ng⋅h/mL) |
|  |  | **Observed** | | **Model Predicted** | |
| Children (Cohort 3) | 3 | 3320 | 17411 | 3618 | 17598 |
| AZL-M 0.66 mg/kg |  |  |  |  |  |
| Children (Cohort 2) | 3 | 2960 | 18691 | 2866 | 17986 |
| AZL-M 20 mg |  |  |  |  |  |
| Children (Cohort 2) | 4 | 3858 | 22555 | 2881 | 21483 |
| AZL-M 40 mg |  |  |  |  |  |
| Children (Cohort 2) | 1 | 2810 | 16055 | 2302 | 16318 |
| AZL-M 60 mg |  |  |  |  |  |
| Adolescents (Cohort 1a) | 6 | 2512 | 17423 | 2557 | 17362 |
| AZL-M 40 mg |  |  |  |  |  |
| Adolescents (Cohort 1a) | 2 | 3245 | 23889 | 3012 | 22975 |
| AZL-M 60 mg |  |  |  |  |  |
| Adults (Cohort 1b) | 9 | 5699 | 40613 | 4839 | 38168 |
| AZL-M 80 mg |  |  |  |  |  |

AUC, area under the plasma concentration-time curve; *C*_max_, maximum plasma concentration
